# Intralesional injection of adipose-derived stem cells reduces hypertrophic scarring in a rabbit ear model

**DOI:** 10.1186/s13287-015-0133-y

**Published:** 2015-08-18

**Authors:** Qi Zhang, Li-Na Liu, Qi Yong, Jing-Cheng Deng, Wei-Gang Cao

**Affiliations:** Department of Plastic and Reconstructive Surgery, Shanghai Ninth People’s Hospital, Shanghai Jiao Tong University School of Medicine, 639 Zhi-Zao-Ju Road, Shanghai, 200011 China; Medical Science & Research Center, Beijing Shijitan Hospital, Capital Medical University, 10 Tie-Yi Road, Beijing, 100038 China

## Abstract

**Introduction:**

Redundant collagen deposition at sites of healing dermal wounds results in hypertrophic scars. Adipose-derived stem cells (ADSCs) exhibit promise in a variety of anti-fibrosis applications by attenuating collagen deposition. The objective of this study was to explore the influence of an intralesional injection of ADSCs on hypertrophic scar formation by using an established rabbit ear model.

**Methods:**

Twelve New Zealand albino rabbits were equally divided into three groups, and six identical punch defects were made on each ear. On postoperative day 14 when all wounds were completely re-epithelialized, the first group received an intralesional injection of ADSCs on their right ears and Dulbecco’s modified Eagle’s medium (DMEM) on their left ears as an internal control. Rabbits in the second group were injected with conditioned medium of the ADSCs (ADSCs-CM) on their right ears and DMEM on their left ears as an internal control. Right ears of the third group remained untreated, and left ears received DMEM. We quantified scar hypertrophy by measuring the scar elevation index (SEI) on postoperative days 14, 21, 28, and 35 with ultrasonography. Wounds were harvested 35 days later for histomorphometric and gene expression analysis.

**Results:**

Intralesional injections of ADSCs or ADSCs-CM both led to scars with a far more normal appearance and significantly decreased SEI (44.04 % and 32.48 %, respectively, both *P* <0.01) in the rabbit ears compared with their internal controls. Furthermore, we confirmed that collagen was organized more regularly and that there was a decreased expression of alpha-smooth muscle actin (α-SMA) and collagen type Ι in the ADSC- and ADSCs-CM-injected scars according to histomorphometric and real-time quantitative polymerase chain reaction analysis. There was no difference between DMEM-injected and untreated scars.

**Conclusions:**

An intralesional injection of ADSCs reduces the formation of rabbit ear hypertrophic scars by decreasing the α-SMA and collagen type Ι gene expression and ameliorating collagen deposition and this may result in an effective and innovative anti-scarring therapy.

## Introduction

After injury in dermal tissue, hypertrophic scars can occur because of abnormal extracellular matrix deposition and remodeling, especially with collagen [1]. This scar tissue is usually raised and inflexible with itching, pain, and redness as a result of an overabundant wound matrix and might give rise to significant cosmetic and functional problems for patients [2]. Today there are all kinds of therapies for hypertrophic scars, including excision, intralesional corticosteroid injection, compression, laser, and interferon injection. However, none of these treatments has been confirmed to be effective in fully avoiding excessive scar tissue formation and regenerating healthy dermal tissue [3, 4]. Hence, it remains a challenge for clinicians to cure hypertrophic scarring.

Major mechanisms during the wound-healing process have already been well studied [5]. Hypertrophic scars occur because of specific factors during the wound-healing process, including inflammation, proliferation, and remodeling. The immune function disorders of T cells and macrophages, accompanied by an extensive inflammatory response, can form void-filling, non-functional tissue and result in the evolvement of scars [6–8]. Reactive oxygen species (ROS) are also a potent driver of deposition of collagen, which is a kind of highly cytotoxic compound secreted by neutrophils for wound sterilization [9, 10]. Transforming growth factor-beta 1 (TGF-β1), a known booster of dermal fibrosis, is a critical collagen-stimulating element in fibroblasts. Besides, TGF-β1 inhibits matrix metalloproteinase (MMP) expression resulting in the accumulation of collagen fibers within the wound sites [11–13]. Myofibroblast differentiation is another intensifier of fibrosis during wound healing. Myofibroblasts, differentiated from fibroblasts in an injury environment, are intended for narrowing the margin of the wounds and accelerating the re-epithelialization by contraction. However, at the same time, they produce tensed, excessive, and irregularly arranged collagen bundles and bring on over-contraction that characterizes hypertrophic scars [14, 15]. Prolonged wound healing usually results in hypertrophic scarring. Consequently, it is very important to ensure the formation of an adequate microvascular network and development into a permanent vascular network during the healing process; otherwise, wound closure will be impaired and hypertrophic scars will occur [16].

During the wound-healing process, any abnormality can have a negative influence on tissue regeneration and contribute to the hypertrophic scar formation. From a therapeutic perspective, medicines that modulate the abnormalities in the period of wound healing may be of benefit in the therapy of scars. According to scientific efforts on many fibrotic diseases, adipose-derived stem cell (ADSC) therapy may be a hopeful method of preventing fibrosis through decreasing inflammation, inhibiting TGF-β1, and favoring tissue regeneration at the wound site [17–19]. Our investigations through the present study show the anti-scaring effect of an intralesional ADSC injection in a rabbit ear hypertrophic scar model.

## Methods

### Rabbit ear hypertrophic scar model

**Fig. 1:**
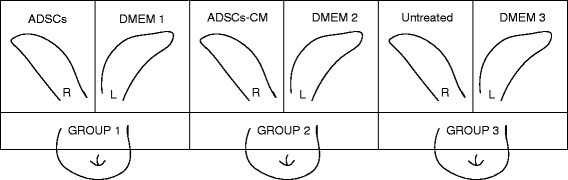
Experiment design. Three groups of rabbits were enrolled. ADSCs, ADSCs-CM, and no treatment were administered to the right ears of the first, second, and third groups, respectively. The left ears of all groups received DMEM injections as an internal control. *ADSC* adipose-derived stem cell, *ADSCs-CM* conditioned medium of the adipose-derived stem cells, *DMEM* Dulbecco’s modified Eagle’s medium, *L* left, *R* right

The rabbit ear hypertrophic scar model as described previously [20] has been well established. Briefly, 12 adult New Zealand albino rabbits (each weighing 2.5 to 3 kg) were given anesthesia under sterile conditions in preparation for wounding based on a protocol approved by the Animal Care and Experiment Committee of the Shanghai Jiao Tong University School of Medicine. Six circular, full-thickness, 1-cm wounds were made to the bare cartilage on the ventral surface of each ear by using a trephine, and the epidermis, dermis, and perichondrium were carefully removed. The wounds were covered with erythromycin eye ointment and cleaned of secretions the next day. The study has excluded samples of infective or necrotic wounds (Fig. 1).

### Preparation of ADSCs and conditioned medium

Four-week-old New Zealand albino rabbits were sacrificed, and groin fat pads were collected, minced, and digested with 0.075 % collagenase type I (Sigma-Aldrich, St. Louis, MO, USA) at 37 °C for 45 min with constant shaking. The cell suspension was centrifuged at 1200×*g* for 10 min. Then the pellet was resuspended and maintained at 37 °C in a humidified atmosphere containing 95 % air and 5 % CO_2_ in regular medium—low-glucose Dulbecco’s modified Eagle’s medium (DMEM), 10 % fetal bovine serum (FBS), 1 % penicillin/streptomycin; Invitrogen, Carlsbad, CA, USA—which was changed every 3 days until 80–90 % confluency was reached. The cells were cultured to the third passage and used for the following steps.

Six-well plates were used for seeding ADSCs (about 5×10^4^ cells/cm^2^) in the third passage. Sixteen hours after seeding, the regular medium was replaced by DMEM containing no FBS. Finally, after incubating for 48 h, supernatant was collected, centrifuged at 300×*g* for 5 min, and filtered through a 0.22-μm syringe filter.

### Identification of ADSCs

Cells were verified by flow cytometry and examined for multiple lineage differentiation as requested by the International Society for Cellular Therapy. For flow cytometric analysis, ADSCs of the third passage were incubated with monoclonal PE-conjugated antibodies for CD34, CD105, HLA-DR, and CD73 or with FITC-conjugated antibodies for CD14, CD90, and CD45 at room temperature for 30 min (BD Pharmingen, San Diego, CA, USA). Isotype control IgG was used to stain the cells as control. The cells were subsequently washed with phosphate-buffered saline (PBS), fixed with 4 % formaldehyde, and analyzed on a FACScan flow cytometer (Becton-Dickinson, San Jose, CA, USA).

For adipogenesis assessment, the cells were incubated in adipogenic medium which included the regular culture medium supplemented with 0.5 mM 1-methyl-3-isobutylxanthine, 1 mM dexamethasone, 10 μl/l insulin, and 100 mM indomethacin (Sigma-Aldrich). The medium was refreshed every 3 days. Adipogenesis was confirmed 21 days later by neutral lipid droplets in the cytoplasm and positive oil red O staining observed in an inverted-phase contrast microscope.

For osteogenic differentiation, we incubated cells at 100 % confluency with an osteoinducing medium containing the regular culture medium further supplemented with 10^−8^ M dexamethasone, 0.3 g/l L−glutamine, 10 mM β-phospherglycerol, and 50 mg/l ascorbic acid (all from Sigma-Aldrich). Calcium deposits, the main characteristic of osteogenesis, were confirmed by positive staining with alizarin red S (Sigma-Aldrich).

### Intralesional injection

ADSCs were harvested at the third passage and labeled with Dil (Invitrogen) in accordance with the instructions of the manufacturer. In brief, ADSCs were washed twice with PBS after being harvested, suspended in Dil dilution (5 μl/ml in DMEM), and incubated at 37 °C for 20 min. After incubation, they were washed twice again and suspended in low-glucose DMEM for injection. Four million ADSCs in 0.2 ml of DMEM were slowly and carefully injected into the center of each lesion from the edge of the wound with a 29-G needle. Similarly, 0.2 ml of ADSCs-CM or DMEM was injected into each lesion in the same way.

### Evaluation of scars

Photos were taken every week to observe any external changes to the wounds since the operation. Ultrasonography of the lesion was detected by an ultrasound machine (MyLabOne, Esaote, Italy) before and 2, 3, 4, and 5 weeks after the surgery to record any internal changes of the scar.

### Histological analysis

Scars were harvested for histologic detection 35 days after the operation. They were bisected and immediately fixed in 10 % formalin. Afterwards, the scars were embedded in paraffin and cut into sections. For scar elevation index (SEI) qualification, sections were stained with hematoxylin and eosin, examined under microscope (Nikon Eclipse E400; Nikon, Tokyo, Japan), and qualified with a digital image analysis system (NIS-Elements Basic Research; Nikon Instech Co., Kanagawa, Japan). The measurement was done twice by a blinded examiner, and averaged values were used. Further evaluation of collagen fiber arrangement was done with Masson trichrome staining.

### Tracing of ADSCs

After being harvested, scar tissues were bisected and fixed in 4 % paraformaldehyde containing 10 % sucrose at 4 °C for 12 h and then 30 % sucrose for 24 h. The specimens were then embedded in OCT compound and stored at −80 °C until use. They were cut at 10 μm, mounted onto SuperFrost-Plus charged slides (Citotest, Jiangsu, China), and washed three times with PBS. After being air-dried for 10 min, they were subjected to 4′,6−diamidino−2−phenylindole (DAPI) (1 μg/ml; Sigma-Aldrich) for nuclear staining and photographed with fluorescence microscopy.

### Total RNA extraction and real-time quantitative polymerase chain reaction

Scar tissues were snap-frozen in liquid nitrogen immediately after harvest. Before total RNA could be obtained, the scar samples were homogenized with an electronic high-speed homogenizer (Scientz, Zhejiang, China). Total RNA was separated with TRIzol reagent (Invitrogen, Life Technologies, Carlsbad, CA, USA) in accordance with the instructions of the manufacturer. The concentration and purity of the total RNA were determined with optical density 260/280 nm measurements (Nanodrop, Isogen, Scotland, UK), and cDNA was generated from the total RNA by reverse transcription with oligonucleotide (dT) primers. The cDNA was diluted in DNase-free water (1:25) and then amplified with a SYBR Green real-time PCR Kit (TaKaRa, Shiga, Japan) with primer pairs for rabbit alpha-smooth muscle actin (α-SMA), collagen type I, and β-actin. The primer pairs were the following:

ACTA2 (National Center for Biotechnology Information (NCBI) Gene ID: 100009271) forward 5′-CAGGGAGTAATGGTTGGAAT-3′ and reverse 5′-TCTCAAACATAATCTGGGTCA-3′, COL1A2 (NCBI Gene ID: 100008997) forward 5′-CCCAACCAAGGATGCACTA-3′ and reverse 5′-CTTGGCCTTGGAGCTCTTATAC-3′, and ACTB (NCBI Gene ID: 100009272) forward 5′-GCTATTTGGCGCTGGACTT-3′ and reverse 5′-GCGGCTCGTAGCTCTTCTC-3′. We used an iCycler Real-Time PCR detection system to perform the gene expression analysis (Applied Biosystems, Foster City, CA, USA). Results were standardized with reference gene β-actin, and the 2^−DDCt^ and fold changes were calculated manually.

### Statistical analysis

Wounds were made, treated, and harvested in a matched fashion with their internal controls. GraphPad Prism 6 (GraphPad Software, Inc., La Jolla, CA, USA) was used to analyze the data. The Student’s *t* test was used to make the comparison of the SEI and gene expression in treated scars and their internal controls. Statistical significance was considered when *P* < 0.05.

## Results

### Identification of ADSCs

**Fig. 2:**
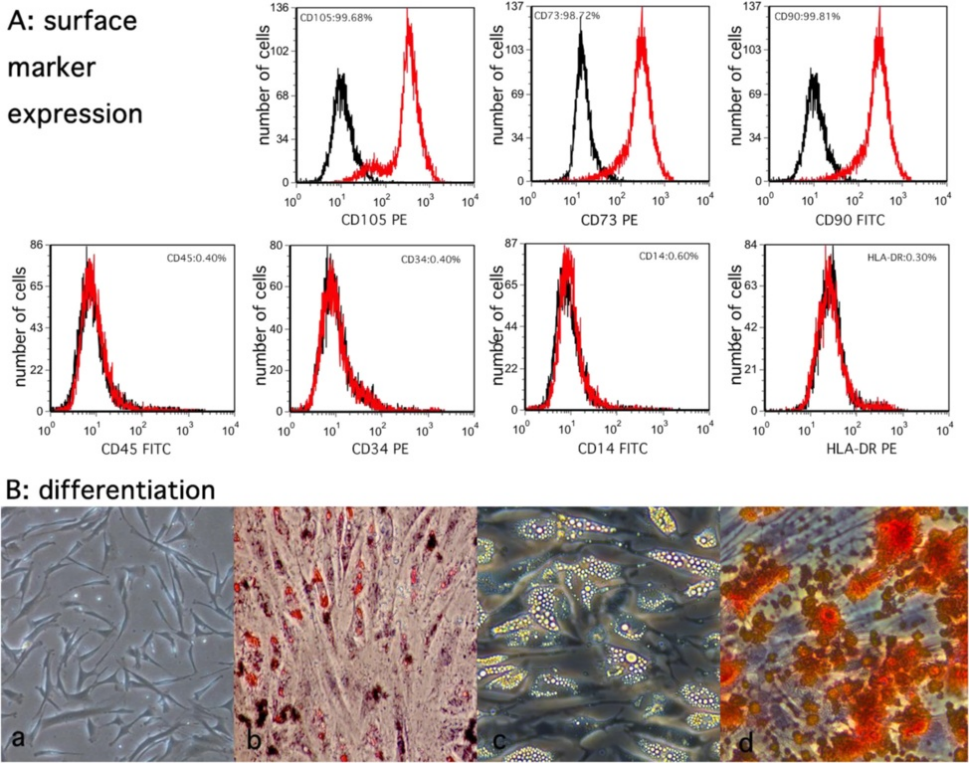
Characterization and differentiation of rabbit ADSCs. **a** Flow cytometry analysis shows that the majority of ADSCs are negative for CD45, CD34, CD14, and HLA-DR and positive for CD73, CD90, and CD105 and this is consistent with the typical mesenchymal stem cell surface marker profile (*black line*: isotype control; *red line*: sample). **b** Cultured human ADSCs display a typical fibroblast-like morphology (**a**) and are able to differentiate between adipogenic and osteogenic lineages in vitro, as demonstrated by positive oil red O staining (**b**), lipid droplet formation (**c**), and alizarin red S staining for calcium deposit formation (**d**). Original magnifications: ×40 (**a**, **b**, **d**) and ×100 (**c**). *ADSC* adipose-derived stem cell

ADSCs exhibited fibroblast morphology and expanded easily when cultured in regular medium in vitro. They were confirmed positive for CD73, CD90, and CD105 and negative for CD34, CD45, CD14, and HLA-DR expression according to flow cytometry analysis of stem cell-related surface markers (Fig. 2a). They can also be successfully trans-differentiated into adipocytes and osteocytes, and this was confirmed by using oil red O staining and alizarin red S (Fig. 2b).

### Both ADSC and ADSCs-CM treatments reduce scar hypertrophy

#### Gross examination

**Fig. 3:**
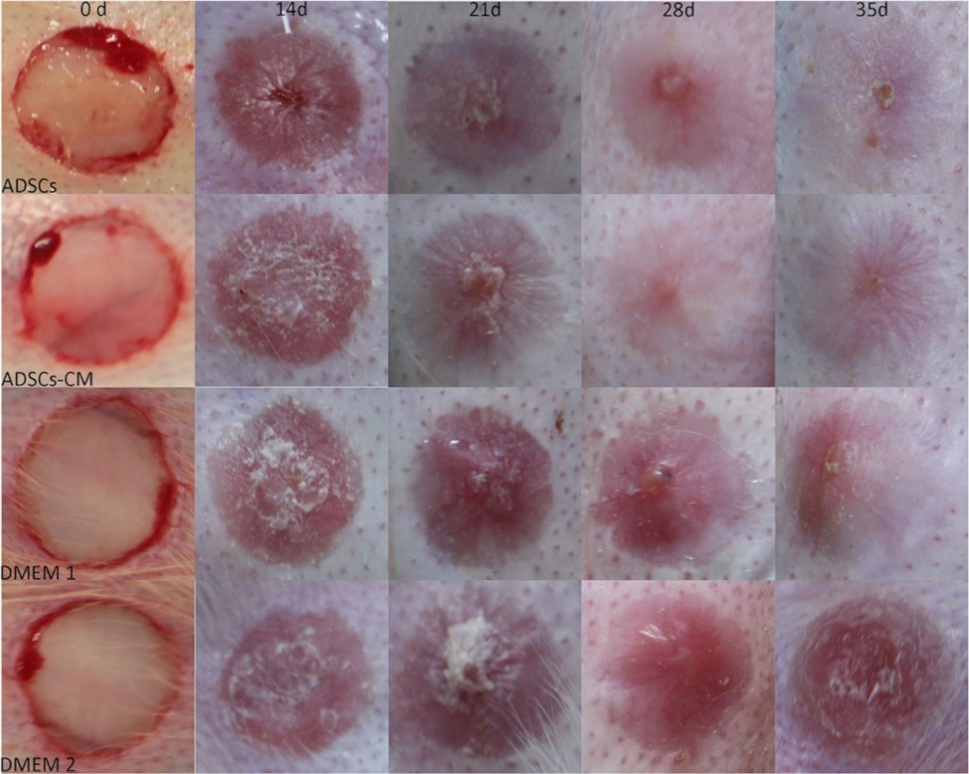
Gross examination images. Both ADSC- and ADSCs-CM-treated scars improved significantly on gross examination and were softer and less visible compared with their internal controls (DMEM 1 and DMEM 2), which gradually became raised, red, and stiff after re-epithelization. *ADSC* adipose-derived stem cell, *ADSCs-CM* conditioned medium of the adipose-derived stem cells, *DMEM* Dulbecco’s modified Eagle’s medium

On postoperative day 14, all wounds were totally re-epithelialized on gross examination. After re-epithelization, a stiff and visibly raised scar gradually formed in both the control and untreated scars. By contrast, a significant improvement of scars, which were less visible and softer, was noticed in both ADSC- and ADSCs-CM-treated scars (Fig. 3).

#### Ultrasonography and calculated SEI

**Fig. 4:**
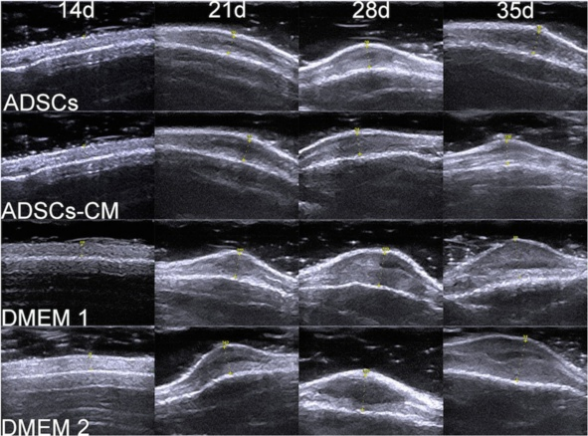
Ultrasonography of the wounds. Ultrasonography shows a gradually growing thickness of the scars in the internal control sides (DMEM 1 and DMEM 2) but almost no change in the ADSC- and ADSCs-CM-treated scars. *ADSC* adipose-derived stem cell, *ADSCs-CM* conditioned medium of the adipose-derived stem cells, *DMEM* Dulbecco’s modified Eagle’s medium

We employed ultrasonography to monitor the changes of wounds. It recorded the accurate thickness of the scar tissue from the epithelium to cartilage and calculated the SEI; this was quite different from traditional methods that measured the total thickness, including the cartilage and tissue below. Ultrasonography of the wounds showed a gradually growing SEI that was finally distinct from and exceeded the surrounding unwounded skin in the control and untreated scars, whereas a slight change was shown in the ADSC- or ADSCs-CM-treated ones (Figs. 4 and 7a).

#### Hematoxylin-and-eosin staining and calculated SEI

**Fig. 5:**
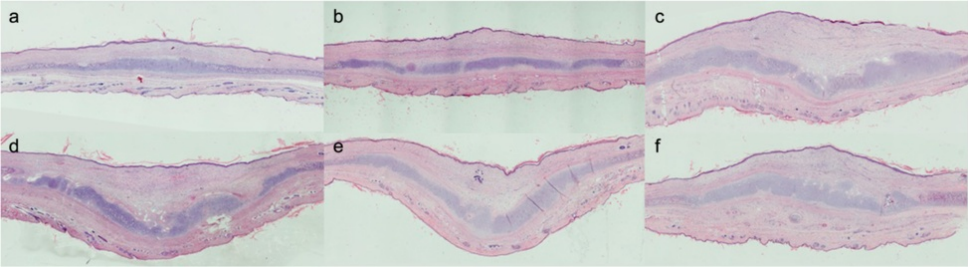
Histologic analysis of wounds. On postoperative day 35, scars treated with ADSCs or ADSCs-CM were even (**a**, **b**), whereas the scars treated with nothing and DMEM (DMEM 1, 2, and 3) were obviously thickened with contraction (**c**, **d**, **e**, **f**). Cells were stained with hematoxylin and eosin. Original magnifications: ×40. *ADSC* adipose-derived stem cell, *ADSCs-CM* conditioned medium of the adipose-derived stem cells, *DMEM* Dulbecco’s modified Eagle’s medium

On postoperative day 35, hematoxylin-and-eosin staining showed that the control and untreated scars were obviously thickened with slight contraction. However, the ADSC- or ADSCs-CM treated ones were flatter and thinner and this is consistent with the gross macroscopic findings (Fig. 5). The SEIs in treated scars of groups 1 and 2 were much lower than their internal controls (group 1: 1.08 ± 0.05 versus 1.93 ± 0.09, *P* < 0.01, n = 24; group 2: 1.33 ± 0.10 versus 1.97 ± 0.11, *P* < 0.01, n = 24). However, there is no significant difference between untreated and DMEM-injected scars in group 3 (group 3: 1.90 ± 0.12 versus 1.94 ± 0.06, *P* > 0.05, n = 24, Fig. 7b).

#### Masson trichrome staining

**Fig. 6:**
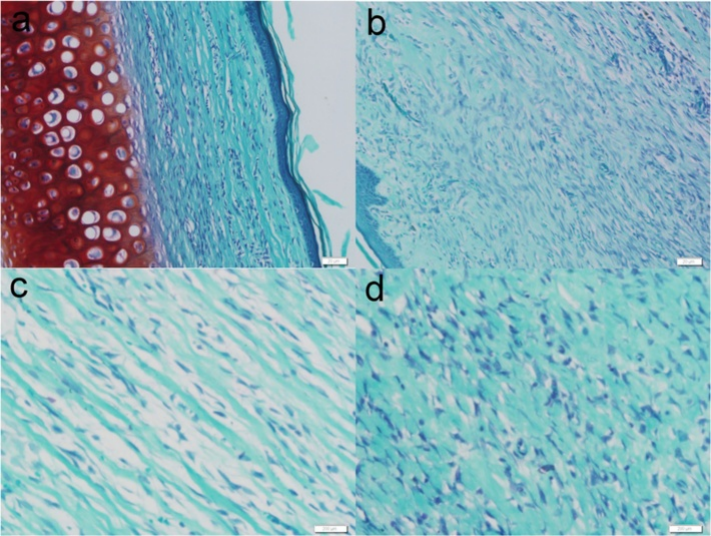
Masson trichrome staining. Masson trichrome staining was used for the evaluation of collagen fiber organization. Collagen fibers were dense and disorderly in the internal control and untreated scars (**b**, **d**). In the ADSC- or ADSCs-CM-treated scars, collagen fibers were regularly arranged (**a**, **c**). Original magnifications: ×200 (**a**, **b**) and ×400 (**c**, **d**). *ADSC* adipose-derived stem cell, *ADSCs-CM* conditioned medium of the adipose-derived stem cells

Masson trichrome staining was used for observation of collagen deposition. On postoperative day 35, collagen fibers were dense and irregularly arranged in the DMEM and untreated scars. Conversely the deposition was eased and collagen fibers were well arranged in the ADSC and ADSCs-CM injection scars (Fig. 6).

#### Detection of gene expression

**Fig. 7:**
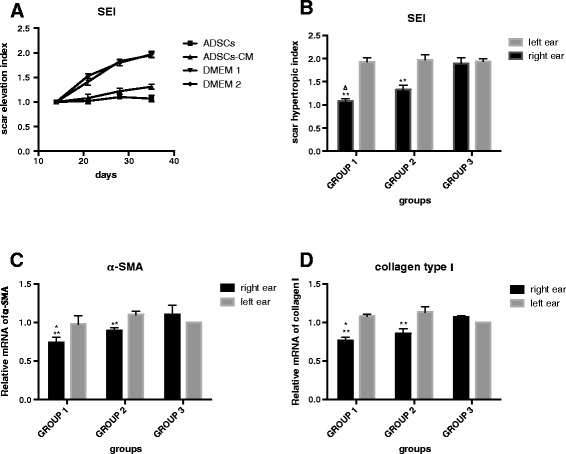
SEI detected by ultrasonography and hematoxylin-and-eosin staining and gene expression analysis. **a** The SEI detected by ultrasonography increased significantly in the internal control scars (DMEM 1 and DMEM 2) in comparison with scars treated with ADSCs and ADSCs-CM on postoperative day 14, 21, 28, and 35. **b** Hematoxylin-and-eosin staining showed that the SEI decreased significantly in scars treated with ADSCs or ADSCs-CM in comparison with their internal controls on postoperative day 35 (44.04 % and 32.48 %, respectively, **P < 0.01 versus internal control). The SEI of ADSC injection scars was much lower than that of ADSCs-CM-treated scars (Δ *P* < 0.01). There was no significant difference between untreated and DMEM injection scars (*P* > 0.05). **c**, **d** The effect of ADSCs and ADSCs-CM on α-SMA and collagen type Ι mRNA expression was measured by quantitative polymerase chain reaction by using the housekeeping gene β-actin as an internal standard control. Both treatments inhibited α-SMA (c) and collagen type Ι (d) mRNA expression in groups 1 and 2 (both **P < 0.01 versus internal control). ADSC treatment resulted in an even lower gene expression of α-SMA and collagen type Ι in comparison with ADSCs-CM injection (*P < 0.05). There was no difference between DMEM injection and untreated scars in terms of α-SMA and collagen type Ι mRNA expression (*P* > 0.05). α-SMA alpha-smooth muscle actin, *ADSC* adipose-derived stem cell, *ADSCs-CM* conditioned medium of the adipose-derived stem cells, *DMEM* Dulbecco’s modified Eagle’s medium, *SEI* scar elevation index

The effect of ADSCs and ADSCs-CM on α-SMA and collagen type Ι mRNA expression was measured by quantitative polymerase chain reaction by using the housekeeping gene β-actin as an internal standard control. Both treatments inhibited α-SMA (Fig. 7c) and collagen type Ι (Fig. 7d) mRNA expression in groups 1 and 2 (*P* < 0.01). There was no difference between DMEM injection and untreated scars in terms of α-SMA and collagen type Ι mRNA expression.

### Vehicles (DMEM) have no effect on scar reduction

Group 3 (right ear: untreated; left ear: DMEM injection) was set up for the detection of the therapeutic effect of DMEM injection. The results show that DMEM injection has no effect on scar reduction. In group 3, the SEI values (Fig. 7b) in the DMEM injection scars were comparable to those in wounds that received no treatment. Also, there is no significant difference of α-SMA and collagen type I gene expression (Fig. 7c, d) between untreated and DMEM-injected scars in group 3. Thus, it also confirmed that DMEM injection did not contribute to the improvement of scars in groups 1 and 2.

### ADSCs were more effective than ADSCs-CM in reducing hypertrophic scars

Both ADSCs and ADSCs-CM can reduce the scar hypertrophy compared with their internal controls. We also found that ADSCs were more effective than ADSCs-CM in further quantitative analysis of the SEI values and mRNA levels. The SEI values were significantly lower in ADSC injection scars of group 1 compared with ADSCs-CM-treated ones of group 2 (Δ*P* < 0.01) (Fig. 7b). Also, there was a decrease of mRNA levels of α-SMA and collagen type Ι in ADSC therapy in contrast to ADSCs-CM treatment (both *P < 0.05) (Fig. 7c, d).

### Viable ADSCs were confirmed in the cell injection group

**Fig. 8:**
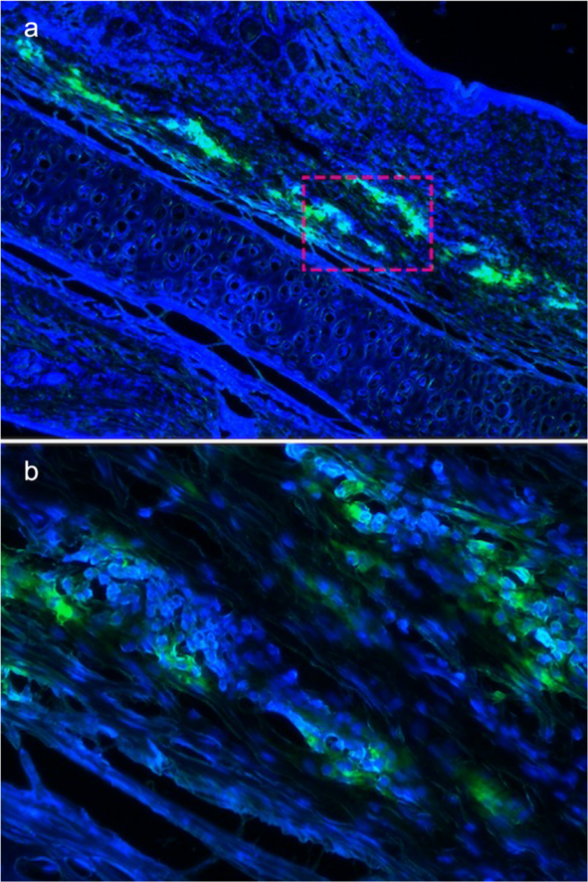
Tracing of viable ADSCs. Viable ADSCs were confirmed in the cell injection group. A large number of Dil-labeled ADSCs were observed (green fluorescence) in the ADSC-injected scar tissue 3 weeks after the initial treatment (**a**, **b**). DAPI was used for nuclear staining. Original magnifications: ×40 (**a**) and ×100 (**b**). *ADSC* adipose-derived stem cell, *DAPI* 4′,6-diamidino-2-phenylindole

Before the injection, ADSCs were marked with Dil, a flourochrome used for tracing live cells. They were detected by frozen sections with a fluorescent microscope after harvesting. We recorded a large number of green fluorochrome-labeled cells with blue-fluorescence nuclei evenly distributed in the ADSC-injected scar tissues 3 weeks after the initial treatment (Fig. 8). Thus, a large number of live cells were confirmed in the ADSC-treated scars, which probably contributed to the inhibition of hypertrophic scars.

## Discussion

Devastating consequences such as organ dysfunction or body disfigurements can result from excess scar formation, which is secondary to surgical or traumatic injuries. However, it has been interestingly observed in several clinical studies that scar tissue from some patients with burn injuries transformed into those histologically similar to normal tissues after undergoing fat transplantation [21–23]. Although this mechanism has not been fully understood, it was conjectured that the performance of stem cells from adipose tissue might give rise to such histologic and clinical scar improvement.

Potential causes for hypertrophic scars revealed by our knowledge of the wound-healing process might contribute to the advance of an effective cure for hypertrophic scar formation [1, 2, 24]. Wound healing is a complex process that consists of three sequential yet superimposed phases: inflammatory, cellular proliferation, and remodeling phases. Hypertrophic scars can occur as a result of an abnormality in the process. Many studies demonstrate that mesenchymal stem cells (MSCs) have an anti-scaring effect by means of favoring wound healing during the process.

Discovered by Zuk et al. in 2001 [25], ADSCs are a kind of MSCs but are more abundant and can be readily acquired, separated, and cultured. They are now widely used as seed cells in tissue engineering as well as in studies promoting wound healing, whitening, anti-aging, and anti-fibrosis [26–30]. Plenty of research on the effect of ADSCs in fibrosis was previously done in animal models or cells. A treatment of injecting ADSCs on injured vocal folds has been performed by one group, which demonstrated the ability of ADSCs to prevent vocal fold atrophy and scarring in a canine animal model because of their multipotential ability in the regeneration of injured vocal folds [17]. Another group studied the influence of ADSC therapy on cardiac remodeling and contractility by using the acute myocardial infarction mouse model and observed great improvement in ventricular remodeling and cardiac function with smaller infarct size and less scar formation. They found that ADSCs can migrate into infarct sites and integrate into scar areas and that some expressed the endothelial marker which might have favored increased vascular density in the injured site [19]. Furthermore, one group found that ADSC treatment can prevent elastosis and fibrotic changes of the tunica albuginea by testing the influence of locally injected ADSCs with a Peyronie’s disease rat model in the active phase [18].

However, the effect of ADSCs on scar formation of the skin has not been studied. Our study investigated the anti-fibrosis effect of ADSCs on the hypertrophic scar formation in vivo. We injected ADSCs and their conditioned medium in the wound on postoperative day 14 after the completion of re-epithelization on a rabbit ear hypertrophic scar model. Both treatments resulted in much lower SEI, more regular collagen arrangement, and decreased expression of α-SMA and collagen type Ι, suggesting that ADSC or ADSCs-CM injections can respectively suppress the formation of hypertrophic scars. The ADSCs-CM contains a lot of growth factors and cytokines secreted by ADSCs, such as IL-10, adrenomedullin, and hepatocyte growth factor (HGF) [31–33]. They have been proven to suppress fibrosis by various mechanisms, including reducing the expression of TGF-β1 and collagen and promoting the expression of MMPs, thus accelerating the turnover of the extracellular matrix [34–37]. The cytokine HGF has also proven the ability of inhibiting myofibroblast differentiation which contributes to the limitation of pro-fibrotic functions of myofibroblasts [15]. During the wound-healing period of proliferation, angiogenesis plays an important role in supplying the fibroblasts with enough nutrients for the formation of an occasional granulation matrix [16]. Also secreted by ADSCs, vascular endothelial growth factor A and basic fibroblast growth factor can offer strong mitogenic cues to accelerate migration, proliferation, and differentiation of microvascular endothelial cells and promote vascular stability [38–41].

Besides, we observed an even lower SEI and gene expression of α-SMA and collagen type Ι in ADSC injection scars than in ADSCs-CM-treated ones. Thus, it can be speculated that ADSCs inhibit hypertrophic scar formation through more than secreted anti-fibrotic factors. Once they have entered the injury site, the MSCs are activated by the inflammatory environment in the wound to start their immunomodulatory function, increasing prostaglandin E_2_ expression and upregulating cyclooxygenase-2 activity [42], which favors wound healing over inflammation and reduces the pro-fibrotic effect that can coincidently exist with immune function disorders of T cells and macrophages in extended inflammation [7, 8]. During the period of wound healing, long exposure to ROS is an intensifier of fibrosis by a mechanism that includes induction of TGF-β1 [9, 43]. Also, MSCs can upregulate inducible nitric oxide when they interact with T cells in a pro-inflammatory environment which can change the ROS/RNS (reactive nitrogen species) balance to stop fibrotic tissues from forming [10].

An additional mechanism by which MSCs may accelerate the cutaneous wound-healing process has been suggested by many studies [44–46]. MSCs could directly take part in the structural regeneration of epidermal and dermal tissues by trans-differentiating into keratinocytes when interacting with native epidermal cells. Recently, one group used a rabbit ear hypertrophic scar model to illustrate that MSCs can attenuate scar formation in a p53-dependent manner [47].

We observed a large number of ADSCs labeled with green fluorochrome in the scar tissue 3 weeks after the initial treatment, confirming the active involvement of ADSCs in wound regeneration. However, we have not come up with an effective method to detect the survival rate of ADSCs, because of the difficulty in counting the number of cells in the scar tissue, and we also failed to trace the final outcome of the ADSCs because of the temporary effect of Dil labeling. Further research with techniques for cell counting in tissues and prolonged cell labeling will be needed to study the survival rate of transplanted ADSCs and whether transplanted ADSCs trans-differentiate into local cells, such as vascular endothelial cells and fibroblasts.

## Conclusions

This study tested the anti-scaring effect of ADSCs and their conditioned medium, in excess of 140 wounds, adopting our well-founded rabbit ear hypertrophic scar model. We have demonstrated that ADSCs can suppress the formation of hypertrophic scars through more than secreting anti-fibrosis cytokines when injected locally in vivo. This is a preliminary study that to our knowledge has not been established by others. Although we need to further investigate ADSCs, it would be novel to adopt them as anti-scarring agents, and there is immense potential for clinical ramifications in the field of hypertrophic scar prevention and treatment.

## Abbreviations

ADSC: Adipose-derived stem cell
ADSCs-CM: Conditioned medium of the adipose-derived stem cells
DMEM: Dulbecco’s modified Eagle’s medium
FBS: Fetal bovine serum
HGF: Hepatocyte growth factor
MMP: Matrix metalloproteinase
MSC: Mesenchymal stem cell
NCBI: National Center for Biotechnology Information
PBS: Phosphate-buffered saline
PCR: Polymerase chain reaction
ROS: Reactive oxygen species
SEI: Scar elevation index
α-SMA: Alpha-smooth muscle actin
TGF-β1: Transforming growth factor-beta 1
